# Safety and tolerability of the antimicrobial peptide human lactoferrin 1-11 (hLF1-11)

**DOI:** 10.1186/1741-7015-7-44

**Published:** 2009-09-08

**Authors:** Walter JFM van der Velden, Thijs MP van Iersel, Nicole MA Blijlevens, J Peter Donnelly

**Affiliations:** 1 Department of Haematology, Radboud University Nijmegen Medical Centre, The Netherlands; 2 Phase-I Clinical Pharmacology Unit, Xendo Drug Development BV, Groningen, The Netherlands

## Abstract

**Background:**

The treatment of patients with haematological malignancies by means of haematopoietic stem cell transplantation (HSCT) is often accompanied by life threatening infections. With emerging antimicrobial resistance there is an increased need for new agents, with a beneficial safety profile. Therefore we evaluated the safety of the promising new antimicrobial peptide human lactoferrrin 1-11 (hLF1-11) in healthy volunteers and patients.

**Methods:**

We undertook a sequential, randomised, double-blind, placebo-controlled study using ascending single (0.005, 0.05, 0.5, 5 mg) and multiple intravenous doses (0.5, 5 mg) in healthy volunteers, and open-label, single intravenous 5 mg doses in autologous HSCT recipients.

**Results:**

Single and multiple doses of hLF1-11 were tolerable up to 5 mg intravenously in healthy volunteers, while 5 mg single dose was tolerable in patients. Elevations in transaminases possibly related to treatment were reversible and not serious.

**Conclusion:**

The new antimicrobial hLF1-11 is well tolerated in healthy volunteers with repeated daily doses up to 5 mg. The side-effect profile is very favourable for an antimicrobial, the only undesirable effect being a possible elevation of transaminases, which may be related to hLF1-11 although the current data do not allow conclusive interpretation of treatment relationship. A lower dose is recommended for the forthcoming multiple dosing studies in HSCT patients.

**Trial registration:**

ClinicalTrials.gov: nct00509938.

## Background

The treatment of patients with haematological malignancies with haematopoietic stem cell transplantation (HSCT) is often accompanied by life threatening complications as a result of the damage caused by the conditioning regimens to the mucosal barrier, and the innate and adaptive, humoral and cellular immune defences [1-3]. Despite many advances in supportive care, transplantation-related morbidity and mortality due to bacterial and fungal infections and uncontrolled inflammation remains high [4,5]. A troublesome fact is the increasing resistance against several important antimicrobial drugs including quinolones, azoles and cephalosporins, making control of bacterial and fungal infections in HSCT a difficult task [6-8]. Therefore, the discovery of a broad array of naturally occurring antimicrobial peptides (AMPs) is interesting, although few AMPs have been studied so far and even less have been studied in clinical settings [9-11].

Human lactoferrin is a natural defence protein present in body fluids and secretions as well as neutrophils [12,13], and has pleiotropic functions including broad spectrum antimicrobial activity, antitumour activity, regulation of cell growth and differentiation, and modulation of inflammatory, humoral and cellular immune responses [14-17]. Levels of lactoferrin are decreased following HSCT [18], contributing to the overall immune deficiency. Correcting this deficit might ameliorate immunity in HSCT recipients [19].

Human lactoferrin 1-11 (hLF1-11) is a lactoferrin derivative being developed for the treatment of bacterial and fungal infections in HSCT recipients. It contains the N-terminal moiety of hLF, consisting of 11 amino acids, that is essential for the antimicrobial and anti-inflammatory activity [14,15]. Preclinical studies have shown promising antimicrobial activity even in the setting of immunodeficiency justifying further investigation for clinical application [20-24]. Being a derivative of a 'natural' human protein, hLF1-11 might have the advantage of fewer side effects and less formation of antibodies and antimicrobial resistance, especially since antimicrobial peptides are unlikely to induce resistance because of the evolutionary difficulty in changing bacterial membrane structure [11].

We report on the first three studies conducted in humans with ascending doses of hLF1-11 in healthy volunteers and in patients receiving autologous HSCT following conditioning with high-dose melphalan (HDM) for multiple myeloma or lymphoplasmocytic lymphoma.

## Methods

### Study design

The 3 studies were conducted sequentially and included a total of 56 subjects (placebo: 12; hLF1-11: 44) as follows. Study 1: single intravenous administration of ascending hLF1-11 doses (0.005, 0.05, 0.5 and 5 mg) in healthy volunteers; study 2: multiple intravenous administration of two ascending hLF1-11 doses (0.5 and 5 mg daily for 5 days) in healthy volunteers; study 3: single intravenous administration of a fixed hLF1-11 dose (5 mg) in patients undergoing an autologous HSCT (Table 1, Additional file 1).

**Table 1:** Entry demographics and dosing schedule.

	**Subjects**	**Mean age (SD), years**	**Mean height (SD), cm**	**Mean weight (SD), kg**	**Mean BMI (SD), kg/m^2^**	**Male/female, n**	**Dosing**	**Dose (mg)**	**Placebo, n**	**hLF1-11, n**	**All, n**
Study 1	Healthy volunteers	24 (5)	185 (6)	79 (9)	23 (3)	32/0	Single	0.005	2	6	8
								0.05	2	6	8
								0.5	2	6	8
								5.0	2	6	8
Study 2	Healthy volunteers	32 (12)	183 (7)	78 (12)	23 (3)	16/0	Multiple	0.5	2	6	8
								5.0	2	6	8
Study 3	HSCT patients	53 (8)	178 (7)	78 (14)	24 (3)	7/1	Single	5.0	-	8	8
									12	44	56

BMI = body mass index; hLF, human lactoferrin; HSCT = haematopoietic stem cell transplant.

### Blinding and subject selection

Studies 1 and 2 were conducted in healthy volunteers, both were randomised, placebo controlled, enrolled 48 volunteers in total (placebo: 12; antimicrobial peptide (AP): 36) (Table 1) and were conducted at the Phase-I Clinical Pharmacology Unit, Xendo Drug Development BV, Groningen, The Netherlands, with prior approval by the appropriate Institutional Review Board (IRB). Entry criteria were similar for studies 1 and 2, namely subjects considered healthy during medical screening by a qualified physician, medical history, physical examination, vital signs, blood and urine evaluations, and 12-lead electrocardiogram (ECG). Age and body mass index (BMI) entry criteria were 18 to 45 years (study 1) and 1 to 65 years (study 2), and 18 to 30 kg/m^2 ^(study 1) and 18 to 28 kg/m^2 ^(study 2), respectively. All volunteers provided written informed consent and the studies were conducted in compliance with current Guidelines on Good Clinical Practice [25]. Enrolment took place between March and April 2005 (study 1) and August and September 2005 (study 2). The aims of both studies were to evaluate the safety, tolerability and pharmacodynamics of intravenous administration of hLF1-11. Safety parameters were adverse events, vital signs, changes in ECG, haematology, clinical chemistry, urinalysis and immunogenicity. Pharmacodynamics evaluations were conducted during multiple dosing (study 2) at baseline, days 1 and 5: cytokine release (interleukin (IL)6, IL10, and tumour necrosis factor (TNF)α) after *ex vivo *stimulation with lipopolysaccharide (LPS) endotoxin in whole blood.

Study 3 was conducted in autologous HSCT recipients. The study was open, and enrolled eight patients (Table 1) between March and October 2006 at the Department of Haematology, Radboud University Nijmegen Medical Centre, The Netherlands, with prior approval by the hospital's IRB. Study conduct was in compliance with current Guidelines on Good Clinical Practice [25]. Entry criteria were males/females aged 18 years or older who were admitted to hospital for autologous HSCT after myeloablative therapy with HDM for multiple myeloma or lymphoplasmocytic lymphoma, BMI <30 kg/m^2^, without serious other pathologies, or history of hepatitis B or C, or HIV infection. The aim of study 3 was to evaluate the safety and tolerability of hLF1-11 given as a single intravenous administration (5 mg) on the day of transplantation. Safety parameters were adverse events, vital signs, changes in ECG, haematology, clinical chemistry, urinalysis, and immunogenicity. Safety and tolerability were evaluated by adverse event reporting, vital signs, changes in ECG, haematology, clinical chemistry, and urinalysis. Adverse events were graded according the to the Common Terminology Criteria for Adverse Events (CTCv3.0, National Cancer Institute, Bethesda, MD, USA) [26].

Blinding in studies 1 and 2 was assured by central allocation of randomisation codes in sealed envelopes, audited on completion of each study and by supply of study medication in indistinguishable form.

For the pharmacodynamics evaluations, cytokine measurements were analysed in study 2 using validated assays for each cytokine once prior to dosing and at six timepoints post dosing (5 min, 30 min, 2 h, 4 h, 8 h, and 24 h) on days 1 and 5, while antibodies against hLF1-11 were measured in all three studies as specific IgG anti-HLF1-11 and IgE anti-hLF1-11 once prior to dosing and at three timepoints post dosing (days 2 and 3, 5 to 8, and 14 and 15) by validated enzyme-linked immunosorbent assay (ELISA), centrally by Xendo Biotech Centre, Groningen, The Netherlands.

Study medication (active and placebo) was supplied by AM-Pharma BV (Bunnik, The Netherlands) as lyophilised powder for solution in normal saline (0.9% NaCl solution) according to dose.

### Statistics, assignment and analysis

Treatment allocation was sequential in all studies, with randomisation lists issued as single blocks of eight (two placebo, six active) for studies 1 and 2, while there was no randomisation in study 3. The intention to treat (ITT) population for safety evaluations was defined in all studies as all randomised subjects who received any medication. Data for studies 1 and 2 were analysed by Xendo Drug Development, Groningen, using SAS version 8.2 (SAS, Cary, NC, USA) and for study 3 by CRM Biometrics GmBH (Bonn, Germany), who also checked the databases for the other two studies. Means, standard deviations, medians, ranges, upper and lower quartiles were calculated, and parameters were listed by subject, summarised and evaluated using descriptive statistics.

## Results

### Subject characteristics and progress through studies

Demographic data for all three studies at entry are displayed in Table 1, with pathological characteristics of patients in study 3 presented in Table 2. One volunteer in study 2, originally allocated to the placebo group (multiple dosing) received one dose of active drug (5 mg) on day 4 due to an administrative error, and his safety data were computed in the active group. All other subjects in the studies received study medication as planned and yielded complete datasets for safety and other analyses.

**Table 2:** Disease characteristics of patients who underwent haematopoietic stem cell transplant (HSCT).

**Patient no.**	**Age (years)**	**Gender**	**Diagnosis, stage^a^**	**Paraprotein type**	**Bence-Jones**	**Prior treatment**	**Status^b^**	**Melphalan dose (mg/kg)**
SC001	61	M	Multiple myeloma, III-A	IgG κ	Not present	VAD, CAD	Partial response	3.92
SC002	62	F	Multiple myeloma, III-A	IgG κ	Present	VAD, CAD	Progressive disease	5.61
SC003	50	M	Multiple myeloma, III-A	Light chain κ	Present	VAD, CAD	Minimal response	5.19
SC004	55	M	Multiple myeloma, III-A	IgG κ, light chain κ	Not present	VAD, CAD	Partial response	5.13
SC005	45	M	Multiple myeloma, III-A	Light chain κ	Present	PAD, CAD	Partial response	6.25
SC006	39	M	Multiple myeloma, III-B	IgG κ	Not present	VAD, CAD	Minimal response	4.37
SC007	54	M	Multiple myeloma, II-A	IgG κ	Not present	Thalidomide, dexamethasone, CAD	Stable disease	5.07
SC008	61	M	Lymphoplasmocytic lymphoma, IV-A	IgG κ, light chain κ	Present	VAD, CAD	Minimal response	5.13

^a^Staging by Durie and Salmon criteria [41]; ^b^status after previous treatment by International Bone Marrow Transplant Registry and Autologous Blood, and Marrow Transplant Registry criteria [42].

ALT = alanine aminotransferase; AST = aspartate aminotransferase; CAD = cyclophosphamide, adriamycin, dexamethasone; PAD = bortezomib, adriamycin, dexamethasone; VAD = vincristine, adriamycin, dexamethasone.

### Safety and tolerability results

The main adverse events from the safety evaluations are presented in Table 3 for studies 1 and 2, and Table 4 for study 3. Overall, intravenous administration of hLF1-11 did not raise safety concerns in either volunteers or patients. During single dosing in volunteers, all events blindly rated as possibly related to treatment were reported once and occurred on placebo and the lowest hLF1-11 dose (0.005 mg), none being reported on the two higher doses. During multiple dosing, the commonest reported events on hLF1-11 were elevations in liver enzymes (alanine aminotransferase (ALT) and aspartate aminotransferase (AST)), which were below twice the upper level of the normal range (ULN; 40 U/l for ALT; 45 U/l for AST) in one volunteer on 0.05 mg and in two volunteers on 5 mg. The third event was below three times the ULN. Detailed analysis of daily measurements of liver enzymes, regardless of levels being reported as adverse events, recorded enzymes levels above the ULN in 1/2 placebo volunteers and in 3/6 volunteers on 0.05 mg dose, while levels above the ULN were recorded in 6/7 volunteers on 5 mg. All but one of the daily measurements were below twice the ULN (one ALT measurement was 127 U/l on day 6; 5 mg dose). All levels were in the normal range within 7 days thereafter.

**Table 3:** Adverse events in healthy volunteers (n = 48).

	**Placebo, n (%)**	**hLF1-11, 0.005 mg, n (%)**	**hLF1-11, 0.05 mg, n (%)**	**hLF1-11, 0.5 mg, n (%)**	**hLF1-11, 5 mg, n (%)**
Study 1 (single dosing):					
Subjects per group	8 (100)	6 (100)	6 (100)	6 (100)	6 (100)
Diarrhoea	-	1 (16.7)	-	-	-
Dizziness	-	-	-	-	1 (16.7)
Epistaxis	1 (12.5)	-	-	-	-
Feeling cold	-	1 (16.7)	-	-	-
Flatulence	-	-	-	-	1 (16.7)
Headache	1 (12.5)	1 (16.7)	-	-	1 (16.7)
Increased appetite	1 (12.5)	-	-	-	-
Phlebitis	-	-	-	-	1 (16.7)
Purpura	-	-	-	-	1 (16.7)
Somnolence	1 (12.5)	-	-	-	1 (16.7)
Study 2 (multiple dosing):					
Subjects per group	3^a ^(100)			6 (100)	7^a ^(100)
ALT increase	-			2 (33.3)	3 (42.9)
AST increase	-			-	1 (14.3)
Dry skin	1 (33.3)			1 (16.7)	-
Hyperhydrosis	-			1 (16.7)	-
Injection site erythaema	1 (33.3)			-	-
Injection site pain	1 (33.3)			-	-
Injection site reaction	-			2 (33.3)	1 (14.3)
Malaise	-			1 (16.7)	-
Nausea	-			1 (16.7)	-

All listed events were rated blindly as possibly related to treatment.

^a^One subject in the placebo group (no. 016) received one dose of hLF1-11 5 mg (day 4) due to administrative error, therefore events were computed in the 5 mg group.

ALT = alanine aminotransferase; AST = aspartate aminotransferase.

**Table 4:** Adverse events in haematopoietic stem cell transplantation (HSCT) patients (n = 8).

**Event**	
All recorded events, n	187
All events possibly treatment related, n	4
Events per patient (min to max), n	5 to 35
Severity (% of events)	
Mild	58
Moderate	25
Severe	17
Non-serious events possibly treatment related, n^a^	4
Supraventricular extrasystoles	1
ALT increased	1
AST increased	1
γ-Glutarate increased	1
Serious adverse events (SAEs), n^b^	4
Heart failure/pulmonary oedema	1
Pulmonary infiltrates	1
Hypoxaemia/respiratory insufficiency	2
Treatment-related SAEs, n	0

^a^All four events occurred in one patient (no. 001); ^b^the four SAEs occurred in two patients (no. 002 and no. 005).

ALT = alanine aminotransferase; AST = aspartate aminotransferase.

In HSCT recipients after conditioning with HDM, as expected, several events were recorded, ranging from 5 to 35 events per patient (Table 4). Four serious adverse events (SAEs) were reported, none of which were considered to be related to hLF1-11, while four non-serious events were reported in one patient, considered possibly related to hLF1-11, and none were reported for the remaining seven patients.

Other clinical laboratory tests (haematology, biochemistry, and urinalysis) did not suggest any treatment-related abnormalities in any of the three studies. No abnormalities in coagulation tests were seen. Haemodynamic tests (blood pressure, heart rate) and 12-lead ECGs (including QT interval) did not yield any treatment-related effects. None of the HSCT recipients developed signs of haemolysis or unexpected cytopoenias and no abnormalities regarding engraftment were seen.

### Pharmacodynamic evaluations

There were no changes in any of the pharmacodynamic evaluations in volunteers during single or multiple dosing or in patients after single dosing. Cytokine measurements in study 2 (healthy volunteers) showed high variability intraindividually and interindividually. Levels of IL10 were undetectable in all samples. Release of IL6 and TNFα on LPS stimulation seemed attenuated with 0.5 and 5 mg; however this was neither clinically nor statistically significant. No antibodies against hLF1-11 were detected (IgG anti-hLF1-11 or IgE anti-hLF1-11) in volunteers or patients.

## Discussion

Bacterial pathogens account for most infections occurring shortly after transplantation during neutropaenia, when mucosal barrier injury is most pronounced. Whilst most patients receive standard antibacterial prophylaxis with fluoroquinolones, the majority of infections are caused by Gram-positive bacteria (65% to 75%), mainly *viridans *group streptococci and coagulase-negative staphylococci as observed in all our patients, and in a minority by Gram-negative pathogens, mostly *Enterobacteriaecae *[27]. The incidence of fungal infections also remains relatively high affecting up to 15% in allogeneic HSCT, with *Candida *species and *Aspergillus fumigatus *predominating [27]. Lactoferrin or derivatives may prove to be a promising versatile class of agents for managing infectious complications that arise from HSCT, because of their broad antimicrobial activity especially in the context of emerging antimicrobial resistance to currently used antimicrobial agents. Additionally, immune modulating and anti-inflammatory properties might attenuate mucosal barrier injury and graft versus host disease in HSCT, although for now this remains speculative [19].

The N-terminal moiety of hLF, in particular the first five amino acids (Gly-Arg-Arg-Arg-Arg), has a high cationic charge allowing binding to negatively charged molecules including microbial products such as lipopolysaccharide (LPS) and CpG motifs of bacterial DNA (CpG-DNA) [28,29]. These interactions result in microbial cell wall disruption and direct microbial killing as a result [30]. Additionally, indirect antimicrobial activity is seen through the intermediary of cells mainly phagocytes cells (polymorphonuclear cells, macrophages) probably as result of opsonisation and probably other not yet fully determined mechanisms [31-33]. Anti-inflammatory and immune-modulatory properties have also been largely related to N-terminal moiety of hLF [14,15,34-36].

hLF1-11, derived from the active N-terminal moiety of hLF, has been tested *in vitro *and *in vivo *showing broad spectrum activity against the pathogens commonly involved in infections after HSCT, both bacterial and fungal, similar to human lactoferrin. The activity in preclinical studies was even superior to that of hLF [37]. *In vivo *animal experiments indicated that hLF1-11 is highly effective against *Staphylococcus aureus*, *Listeria monocytogenes *and antibiotic resistant *Acinetobacter baumannii*, reducing bacterial loads in infected organs by 2 to 3 log [20,23,24,37]. Furthermore, hLF1-11 was effective against invasive infections from fluconazole resistant and fluconazole sensitive *Candida *species even in neutropaenic and lymphopaenic mice [21,22,38]. Regarding immune-modulatory properties, as for the antimicrobial activity, the activity of hLF1-11 is expected to be similar albeit more potent than those reported for hLF, although this has to be studied in more detail.

In this study, for the first time, the antimicrobial peptide hLF1-11 has been tested in healthy volunteers and autologous HSCT recipients. In both populations the drug was well tolerated with few possibly related side effects. The reported events consisted predominantly of discomfort at the injection site, commonly reported with intravenous drugs. No changes on electrocardiography and in particular no increase in QT interval was seen. All adverse events were mild in intensity, reversible without clinical sequel, not necessitating any intervention. No signs of cytotoxicity were seen, consistent with earlier *in vitro *data [39], and in HSCT recipients no changes in engraftment occurred. Pharmacodynamic evaluation revealed no apparent changes in the cytokine profiles, suggesting that the immune-modulatory effects of hLF1-11 do not result in an unexpected increase in proinflammatory responses.

**Figure 1:**
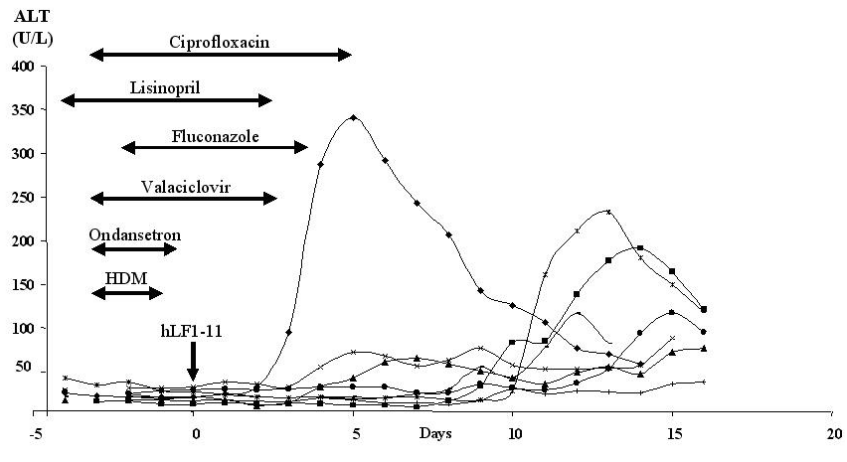
**Serum alanine aminotransferase (ALT) levels in haematopoietic stem cell transplantation (HSCT) patients**. The drugs depicted in figure are the drugs used by the patient experiencing elevated transaminases. day 0 = day of haematopoietic stem cell transplantation; HDM = high-dose melphalan.

During multiple dosing, elevations in transaminase levels were detected and considered related to the study drug in five subjects, because use of other drugs or alcohol was not allowed during the study, although elevations were observed on placebo as well. Similar abnormalities were observed in one patient receiving the 5 mg dose, followed by spontaneous and complete recovery. According to the CTCv3.0 [26], the maximum elevation was observed once in one patient and was moderate (grade 2/3). Since this patient was receiving a number of other drugs known to affect transaminases (Figure 1), this occurred after single dose and no effects on transaminases had been reported during single dosing in healthy volunteers but mild elevations had been recorded after multiple dosing, we considered this event as possibly related to treatment. A safety advisory board (independent of investigators) also evaluated the liver enzyme results of all studies and could not establish a definite relationship between hLF1-11 and elevated transaminases, although it did not rule out such an effect and advised that a lower dose (0.5 mg) should be used in the next multiple dosing study in patients, with close monitoring of liver parameters. This lower dose is expected not to interfere with the antimicrobial activity of hLF1-11, shown in preclinical data to be active in the μg/kg range [22]. Moreover, a recent study emphasised that even at very low concentrations AMPs with strong membrane-binding activity could disrupt bacteria by reaching higher membrane-bound concentrations than intuitively expected [40].

One important evaluation that is missing in our studies is the pharmacokinetics of hLF1-11, as to date the detection of the peptide is impossible to quantify in humans, which makes safety evaluations all the more important. Inability to measure hLF1-11 in plasma is partly due to the doses being so small (0.005 to 5 mg) that the concentrations in plasma are undetectable by usual methods. In animals, ^99m^Tc-labelled hLF1-11 was rapidly removed from the circulation mainly via the liver and to a lesser extent via the kidneys. The quantity of ^99m^Tc-labelled peptide in the liver remained stable during the first hour after injection, while the plasma half life (in mice) was estimated at approximately 30 min, yet this may be a underestimation since some of the peptide may be receptor bound and an unknown amount may be quickly metabolised. Studies are ongoing with internally labelled (C_14 _and tritium) peptide to determine distribution and metabolism in animals, while the quantification in humans remains undetermined. Nevertheless, the results of this new antimicrobial in preclinical studies merit further investigations on the applicability of hLF1-11 in humans, particularly in patients with haematological malignancies, and were the motivation for the current studies.

## Conclusion

The new antimicrobial drug hLF1-11 was well tolerated in healthy volunteers with repeated daily doses up to 5 mg. Owing to elevations in transaminase levels being possibly related to treatment caution is warranted in further studies, although this potential effect was regarded not clinically serious and reversible without intervention in all cases to date. Nevertheless, as a precaution, further testing will be conducted with a lower dose of 0.5 mg in the forthcoming multiple dosing study in HSCT patients.

## Competing interests

Other than the funding sources the authors have no financial or non-financial competing interests to declare.

## Authors' contributions

WvdV carried out the clinical study in patients, gathered and analysed the data, wrote the manuscript, and read and approved the final version of the manuscript. TvI carried out the study in healthy volunteers, gathered and analysed the data, and read and approved the final version of the manuscript. NB carried out the clinical study in patients, gathered and analysed the data, wrote the manuscript, and read and approved the final version of the manuscript. JPD carried out the clinical study in patients, gathered and analysed the data, and read and approved the final version of the manuscript.

## Pre-publication history

The pre-publication history for this paper can be accessed here:



## Supplementary Material

Additional file 1 **Patient flow charts.** Click here for file
