# Supplementary material for: Safety and tolerability of the antimicrobial peptide human lactoferrin 1-11 (hLF1-11)
Source: BMC Med. 2009 Sep 8;7:44. doi: 10.1186/1741-7015-7-44 (PMC2746231; doi:10.1186/1741-7015-7-44)
Supplement: Additional file 1 — Patient flow charts. [file 1741-7015-7-44-S1.doc]

**Patiënt flow charts:**

Study 1: healthy volunteers:

- N=8 placebo.
- N=24 hLF1-11.

All patients completed the study.

Study 2: healthy volunteers.

- N=4 placebo.
- N=12 received hLF1-11.

All patients completed the study. One patient in the placebo arm accidentally received hLF1-11 and crossed over to the hLF1-11 group: giving N=3 placebo and N=13 hLF1-11 in the final analysis.

Study 3: open label safety study in patients.

- N=8 patients enrolled. All completed the study.
